# Reply to Comment on Cobo-Cuenca, A.I.; Laredo-Aguilera, J.A.; Rodríguez-Borrego, M.-A.; Santacruz-Salas, E.; Carmona-Torres, J.M. Temporal Trends in Fecal Occult Blood Test: Associated Factors (2009–2017). *Int. J. Environ. Res. Public Health* 2019, *16*, 2120

**DOI:** 10.3390/ijerph16245014

**Published:** 2019-12-10

**Authors:** Ana Isabel Cobo-Cuenca, Jose Alberto Laredo-Aguilera, Maria Aurora Rodríguez-Borrego, Esmeralda Santacruz-Salas, Juan Manuel Carmona-Torres

**Affiliations:** 1 Facultad de Fisioterapia y Enfermería de Toledo, Universidad de Castilla la Mancha (UCLM), 45071 Toledo, Spain; anaisabel.cobo@uclm.es (A.I.C.-C.); Juanmanuel.carmona@uclm.es (J.M.C.-T.); 2 Grupo de Investigación Multidisciplinar en Cuidados (IMCU), UCLM. Av. Carlos III s/n. 45071 Toledo, Spain; esmeralda.santacruz@uclm.es; 3 Instituto Maimónides de Investigación Biomédica de Córdoba (IMIBIC), 14004 Córdoba, Spain; en1robom@uco.es; 4 Facultad Ciencias de la Salud de Talavera de la Reina, Universidad de Castilla la Mancha (UCLM), 13071 Ciudad Real, Spain; 5 Departamento de Enfermería, Universidad de Córdoba (UCO), 14004 Córdoba, Spain; 6 Hospital Universitario Reina Sofía, 14004 Córdoba, Spain

In this study [1], we used data recollected by the National Institute of Statistics (NIS) of Spain and the Spanish Ministry of Health, Social Services, and Equality (SMHSE).

The European Health Survey in Spain (EHSS) and National Health Survey (NHS) were carried out through personal interviews. Interviews were performed by the NIS and SMHSE using probabilistic multistage sampling with stratification of first- (municipalities) and second-stage (sections) units, with the final units (individuals) selected by random routes and sex- and age-based quotas. The data obtained from these surveys are available in the NIS and SMHSE web [1,2,3,4] in the form of anonymized microdata.

The aims of our study [1] were to determine the prevalence and temporal trends of the fecal occult blood test (FOBT) in people aged 50–69 years, and to determine the sociodemographic profiles and the associated variables with testing FOB.

We used data from the European Health Survey in Spain (EHSS) in 2009 [2] and 2014 [3], and from the National Health Survey (NHS) from 2011/12 [4] and 2017 [5], available in:

Methodology 2009: https://www.ine.es/metodologia/t15/t153042009.pdf [2]
Methodology 2011/12: https://www.ine.es/metodologia/t15/t153041912.pdf [4]
Methodology 2014: https://www.ine.es/metodologia/t15/t153042014.pdf [3]
Methodology 2017: https://www.ine.es/metodologia/t15/t153041917.pdf [5]

Table 3 shows the evolution of performance of the FOB test in the 2009–2017 period in each Spanish Autonomous Community [1].

In this respect, and as explained in our manuscript [1], in 2017, there were many communities in which the implementation of the screening program was recent. Thus, these communities showed a high percentage of FOB performance compared to the rates of those same communities in years prior to the surveys.

In communities such as the Cantabria, País Vasco, and La Rioja, although what is observed is the maintenance over time of the indices used to carry out the test, the data for 2014 show an increase with respect to previous years.

Nevertheless, if, in the Autonomous Community of the País Vasco, instead of valuing the percentages (FOB performance of that community over the years), the absolute frequencies are valued, we observe how there has been an increase in the number of persons who have performed FOBT in the last two years [1].

The purpose of Table 3 was to show the growing trend of FOB testing in the different Spanish autonomous communities during the period indicated. This table shows how the performance of the test has increased unequally within each community over the years. As referred to in our manuscript [1], this inequity may be due to the gradual and uneven implementation of screening programs.

Although the effectiveness of each programme cannot be assessed either with our study [1] in general or with Table 3 in particular, such assessments were not the purpose of our study. Rather, we determine the prevalence and temporal trend of FOBT in people aged 50–69 years and determine the sociodemographic profiles associated with the performance of FOB.

In the Health Survey conducted in the País Vasco in 2018 [6], which was carried out with a stratified sample of 12,071 people surveyed with a participation rate of 79% among those aged over 16, when stratified by age and by FOB test, 89.9% of those surveyed received the letter of invitation, and of these, 89% of the people who received the letter of invitation to perform the test also received the faecal occult blood sample test.

In this respect, we must emphasize that our study not only reflects data on those invited to take the test, but also on those not invited to take part in the screening, i.e., those who participated voluntarily based on their own decisions. Thus, there are differences between our study [1] and the one referred to from the País Vasco [6].

Another aspect of the data to be taken into account in the comparison between both studies (as far as the Basque Community is concerned), ours [1] and that of País Vasco [6], and which may explain the differences between the two, is the date of completion. The Basque Survey [6] was conducted from October 2017 to May 2018, while the data we used in our study [1] from the National Health Survey (NHS) (2017) were collected from October 2016 to October 2017 [4].

In our manuscript, and to highlight the rigor of our research, we must note that in the discussion section, we have taken into account and referenced the way in which screening is carried out in the different health services of the different autonomous communities, including the Basque community.

Likewise, in our article [1], we refer to the fact that screening has been unequally implemented in the different communities: “In 2017, the CRC screening had been implemented in 11 autonomous communities and was being introduced in another five. Now (2018), the rest of the communities have just implemented the colorectal cancer screening (except Ceuta and Melilla). This unequal implementation was due to each region having one public health system that is managed by a different regional government, even though the health system in Spain is public”.

In view of the above, we do not understand the opinions expressed by Mrs. Portillo, given that our article [1] is based on evidence obtained from data provided by state institutions: The National Health Survey (NHS) [4,5] and the European Health Survey in Spain (EHSS) [2,3].

However, we will gladly respond to Mrs. Portillo and the editorial team if they have any other questions that we can answer and/or explain.
